# The DESiGN trial (DEtection of Small for Gestational age Neonate), evaluating the effect of the Growth Assessment Protocol (GAP): study protocol for a randomised controlled trial

**DOI:** 10.1186/s13063-019-3242-6

**Published:** 2019-03-04

**Authors:** Matias C. Vieira, Sophie Relph, Andrew Copas, Andrew Healey, Kirstie Coxon, Alessandro Alagna, Annette Briley, Mark Johnson, Deborah A. Lawlor, Christoph Lees, Neil Marlow, Lesley McCowan, Louise Page, Donald Peebles, Andrew Shennan, Baskaran Thilaganathan, Asma Khalil, Jane Sandall, Dharmintra Pasupathy, on behalf of the DESiGN Collaborative Group, Peter Brocklehurst, Susan Tebbs, Caroline Doré, Paul Seed, Louisa Delaney, Janet Cresswell, Sarah Petty, Bini Ajay, Beverley Wright, Hannah O’Donnell, Melissa Howard, Emma Wayman, Paula Galea, Mandish Dhanjal, Elisa Iaschi, Vanessa Hodge, Hiran Samarage, Sunder Chita, Raffaele Napolitano, Iris Tsikimi, Fiona Ghalustians, Spyros Bakalis, Simona Cicero, Elisabeth Peregrine, Lyndsey Smith, Deepa Janga, Renata Hutt, Edwin Chandraharan

**Affiliations:** 1 Department of Women and Children’s Health, School of Life Course Sciences, Faculty of Life Sciences and Medicine, King’s College London, Women’s Health Academic Centre KHP, 10th Floor North Wing, St. Thomas’ Hospital, Westminster Bridge Road, London, SE1 7EH UK; 2 Centre for Pragmatic Global Health Trials, Institute for Global Health, University College London, Gower Street, London, WC1E 6BT UK; 3 Faculty of Health, Social Care and Education, Kingston and St. George’s University, 6th Floor, Hunter Wing, Cranmer Terrace, London, SW17 0RE UK; 4 The Guy’s and St Thomas’ Charity, 9 King’s Head Yard, London, SE1 1NA UK; 5 Department of Surgery and Cancer, Imperial College London, Kensington, London, SW7 2AZ UK; 6 Population Health Science, Bristol Medical School, University of Bristol, Bristol, BS8 2BL UK; 7 Bristol NIHR Biomedical Research Centre, Bristol, BS8 2BL UK; 8 UCL Institute for Women’s Health, University College London, Gower Street, London, WC1E 6BT UK; 9 Faculty of Medical and Health Sciences, University of Auckland, Victoria Street West, Auckland, 1142 New Zealand; 10 West Middlesex University Hospital, Chelsea and Westminster Hospital NHS Foundation Trust, Twickenham Road, Isleworth, TW7 6AF UK; 11 Fetal Medicine Unit, St George’s University Hospitals NHS Foundation Trust, Blackshaw Road, London, SW17 0QT UK; 12 Molecular and Clinical Sciences Research Institute, St George’s, University of London, Cranmer Terrace, London, SW17 0RE UK

**Keywords:** Small-for-gestational-age foetus, Customised growth centiles, Stillbirth, Implementation research, Health economics

## Abstract

**Background:**

Stillbirth rates in the United Kingdom (UK) are amongst the highest of all developed nations. The association between small-for-gestational-age (SGA) foetuses and stillbirth is well established, and observational studies suggest that improved antenatal detection of SGA babies may halve the stillbirth rate. The Growth Assessment Protocol (GAP) describes a complex intervention that includes risk assessment for SGA and screening using customised fundal-height growth charts. Increased detection of SGA from the use of GAP has been implicated in the reduction of stillbirth rates by 22%, in observational studies of UK regions where GAP uptake was high. This study will be the first randomised controlled trial examining the clinical efficacy, health economics and implementation of the GAP programme in the antenatal detection of SGA.

**Methods/design:**

In this randomised controlled trial, clusters comprising a maternity unit (or National Health Service Trust) were randomised to either implementation of the GAP programme, or standard care. The primary outcome is the rate of antenatal ultrasound detection of SGA in infants found to be SGA at birth by both population and customised standards, as this is recognised as being the group with highest risk for perinatal morbidity and mortality. Secondary outcomes include antenatal detection of SGA by population centiles, antenatal detection of SGA by customised centiles, short-term maternal and neonatal outcomes, resource use and economic consequences, and a process evaluation of GAP implementation. Qualitative interviews will be performed to assess facilitators and barriers to implementation of GAP.

**Discussion:**

This study will be the first to provide data and outcomes from a randomised controlled trial investigating the potential difference between the GAP programme compared to standard care for antenatal ultrasound detection of SGA infants. Accurate information on the performance and service provision requirements of the GAP protocol has the potential to inform national policy decisions on methods to reduce the rate of stillbirth.

**Trial registration:**

Primary registry and trial identifying number: ISRCTN 67698474. Registered on 2 November 2016.

**Electronic supplementary material:**

The online version of this article (10.1186/s13063-019-3242-6) contains supplementary material, which is available to authorized users.

## Background

The rate of stillbirth in England and Wales has declined recently from 5.7/1000 births in 2003 to 4.7/1000 in 2013 [1] but the United Kingdom (UK) rate remains amongst the highest in developed countries [2, 3]. Reducing stillbirth is a national priority [4–6]. It has been estimated that up to 57% of babies who die in utero are small for gestational age (SGA) and 9% have placental insufficiency. However, rates are dependent upon which perinatal classification system is used (32.6–57%) [7–9].

The use of routine screening achieves antenatal detection of SGA in approximately 20–25% of cases [10–13]. In the UK, routine screening involves fundal-height measurements in low-risk pregnancies and serial growth scans in high-risk pregnancies; however, there is variation in risk factors considered and frequency of assessment. Since the publication of the National Health Service (NHS) England ‘Saving Babies’ Lives Care Bundle’ in 2017, there has been a move towards consistency on risk factor stratification [5]. Other strategies, such as universal third-trimester ultrasound, could increase the antenatal detection of SGA to 57% for nulliparous women with singleton pregnancies [13]. A population-based study has reported that antenatal detection of SGA may halve the risk of stillbirth through appropriate antenatal surveillance and timely delivery [10]. This suggests that improvements in the detection of SGA infants could potentially result in further reduction in the incidence of stillbirths.

SGA is traditionally defined as weight below the 10th centile for gestational age and sex according to population references [14, 15]. The development of population-based centiles is an improvement over the use of estimated fetal weight or absolute birthweight to characterise abnormalities of fetal and infant growth. This is exemplified by a 2000-g neonate which will be considered small at term (37–42 completed gestational weeks) but normally grown at 32 weeks. SGA by population birthweight references account only for the physiological effects of gestational age and fetal sex, and it has been hypothesised that there are additional physiological maternal and fetal characteristics that may affect fetal growth [16]. Some foetuses, currently defined as SGA, may be appropriately grown for maternal constitution (i.e. the constitutionally small foetus). Other foetuses with impaired growth may still fall within the normal range by population references, particularly if genetically predetermined to be at higher centiles.

The concept of customised fetal growth standards attempts to address these issues and is based on three principles: (1) individualised (adjusted for physiological factors that affect birth weight – maternal height, weight, ethnicity, parity, fetal sex and gestation at delivery); (2) optimised growth potential (excluding pathological factors affecting the weight standard such as smoking and diabetes) and (3) use of fetal growth curves, derived from normal pregnancies [17]. International studies have demonstrated that the use of customised standards identifies additional SGA foetuses, which were not otherwise identified by population standards [18–22]. Infants who are SGA only by customised standards are at increased risk of adverse outcomes, including stillbirth [18]. These studies have also suggested that foetuses that are considered SGA by population references, but not by customised standards, have outcomes similar to those of appropriately grown foetuses [18–22]. A non-randomised study of the use of customised charts for fundal-height measurement and estimated fetal weight demonstrated an increase in antenatal detection of small babies (47.9% vs. 29.2%, odds ratio 2.23; 95%CI 1.12–4.45) when compared to routine care, but did not demonstrate differences in other key perinatal outcomes, including for stillbirth (five vs. four stillbirths: odds ratio 1.14 (95%CI 0.30–4.25) [23]. Implementation of customised charts has also been explored observationally in Australia where a doubling in the detection rate of SGA was demonstrated in nulliparous women, when compared with historical controls [24].

The Growth Assessment Protocol (GAP) is a programme developed by the Perinatal Institute that consists of the use of gestation-related optimal weight (GROW) customised charts, alongside a schedule of antenatal risk assessment for SGA, management protocols for suspected SGA foetuses, audit tools and training [17]. GROW utilises a systematic method of measurement, achieved through standardised training and accreditation, with measurements plotted onto customised fundal-height charts. Estimated fetal weights from ultrasound assessment are also plotted onto these customised fetal growth charts when growth scans are performed. The risk assessment and management protocols were adapted from the Royal College of Obstetricians and Gynaecologists (RCOG) Green-top Guideline on Investigation and Management of the Small-for-Gestational-Age Foetus [25]. The audit tool is aimed at identifying missed cases and assessing the reasons for failure to antenatally recognise the SGA foetus.

The use of the GAP/GROW programme has expanded since its development and is now implemented in 121 (76%) UK Trusts [26]. A study in the UK compared regions with high uptake to regions with low GAP uptake during the period 2008 to 2012, using stillbirth data from the Office for National Statistics’ records [1]. Overall, there was a 22% lower stillbirth rate in the high-uptake regions during the period analysed, compared to static rates in the low-uptake areas, although it should be noted that the low-uptake regions had lower rates to begin with (4.86 stillbirths/1000 births vs. 5.63/1000 births in the high-uptake regions) and ecological comparisons such as these cannot be assumed to reflect an effect of GAP and may instead reflect a number of changes over time [23]. For example, the Mothers and Babies: Reducing Risk through Audits and Confidential Enquiries across the UK (MBRRACE-UK) Perinatal Mortality Surveillance Report (2013) confirms a gradual decline in stillbirth rates across the UK over a similar period (the decade up to 2013), and attributes this to Sands (the Stillbirth and Neonatal Death charity) raising awareness of a number of initiatives designed to reduce stillbirth rates, and/or to new guidance from the UK Royal College of Obstetricians and Gynaecologists in redefining which stillborn babies require registration (i.e. not those known to have died in utero prior to the end of the 24th gestational week) [3].

It is widely recognised that the highest level of evidence for intervention effectiveness is obtained from randomised controlled trials (RCTs), and a Cochrane review has highlighted that these are currently lacking in this area [27]. A RCT to accurately assess the GAP programme is, therefore, imperative and timely. Furthermore, there is a paucity of data on the effect of GAP on other maternal and neonatal outcomes such as caesarean section rates, induction of labour, gestational age at delivery or on the use of clinical resources including ultrasound scan appointments, neonatal intensive care unit (NICU) admission and lengths of maternal or neonatal stay in hospital. These outcomes may have a significant health-economic impact which must also be balanced against any improvement in perinatal outcomes (stillbirth, early neonatal death, serious neonatal morbidity).

We propose a cluster RCT to evaluate the GAP programme as a strategy for improving the antenatal detection of SGA, including an evaluation of the GAP implementation and a health economic assessment. This protocol (version 7.0, dated 18 January 2018) has been written and reported according to the Standard Protocol Items: Recommendations for Interventional Trials (SPIRIT) guidance and Checklist [28] (see Additional file 1: Table S1-S2).

## Methods/design

### Study objectives

The objectives are to: (1) determine whether implementation of the GAP programme will result in an improved detection of SGA by ultrasound; (2) investigate the effect of the intervention on short-term maternal and neonatal outcomes; (3) estimate the impact of GAP on clinical service provision and health economics and (4) assess fidelity and quality of implementation, acceptability and identify contextual factors associated with variation in the effect of GAP.

### Trial design and setting

**Fig. 1:**
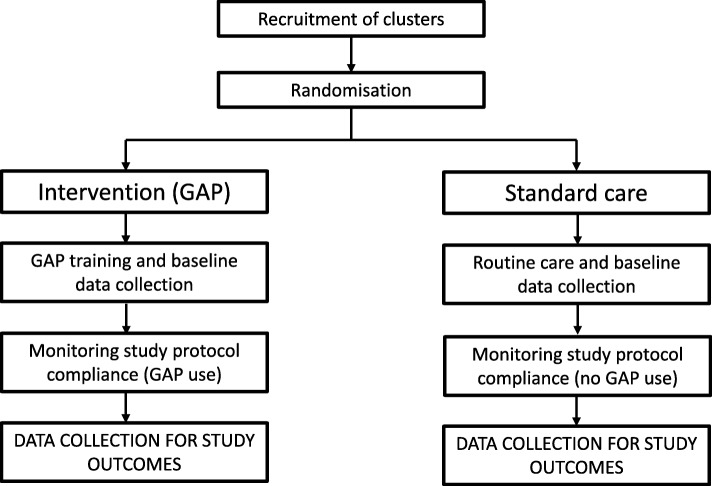
Diagram of expected procedures in participating clusters in the intervention and standard care arms

A cluster RCT will compare the detection rates of SGA in hospitals randomised to the GAP programme to those randomised to standard care (local policy for screening and detection of abnormal fetal growth); see Fig. 1. Maternity units (some containing more than one site) were approached on the basis that they were willing to introduce GAP into their unit and were willing to be randomised to the intervention (immediate implementation of GAP) or standard care (implementation delayed at least until the trial ends). Maternity units randomised to standard care agreed that they would continue with current practice until the completion of the data collection period, at which point they will have the opportunity to implement GAP. Each UK Trust within the study forms a cluster. We intend to test superiority of the GAP in the antenatal detection of SGA by ultrasound.

A cluster RCT design has been chosen due to the nature of the intervention. The GAP programme requires culture change and retraining of multidisciplinary maternity care staff; it is, therefore, not feasible to partially implement GAP in a hospital and randomise individuals to either study arm. The RCT was planned with the majority of the sites in London, since uptake of the GAP programme in this region was low at the start of the trial and the London maternity network had recommended the use of GAP regionally as a strategy to reduce stillbirth rates which were higher than the national average (5.3/1000 in 2013 compared to 4.8/1000 in 2013 in England and Wales [1]).

**Fig. 2:**
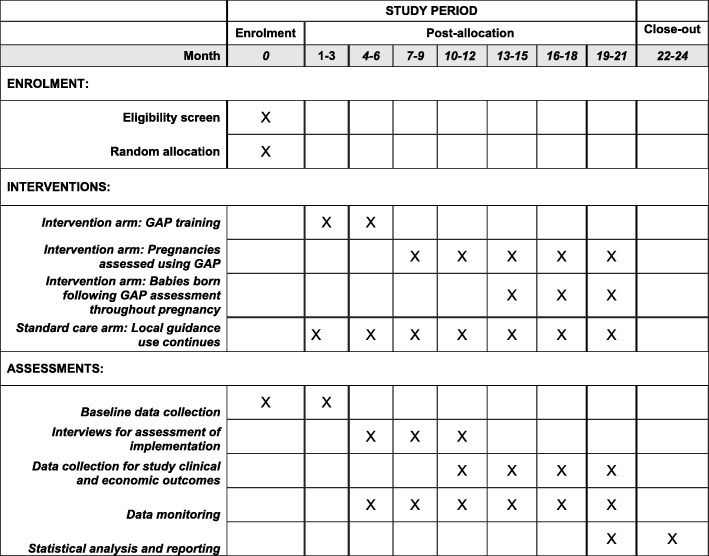
Standard Protocol Items: Recommendations for Interventional Trials (SPIRIT) table for the timeline of study enrolment, intervention and assessment

This is a pragmatic trial for which the intervention will be introduced by the clinical care team with the support of the Perinatal Institute (the only providers of training in GAP/GROW). This approach captures the reality of introducing a complex intervention into clinical practice in these sites. See Fig. 2 for the trial timeline of enrolment, intervention and assessment.

### Eligibility criteria

Eligibility criteria are based on the characteristics of the maternity unit which is the unit of randomisation and not on individual women. Data from all women who give birth within an eligible maternity unit will be included within the study unless individuals specifically opt out of the study. Maternity units that have already fully implemented GAP were not eligible. Data from women with multiple pregnancies or fetal congenital abnormalities will be excluded from the analysis.

### Interventions

#### Description and components

**Fig. 3:**
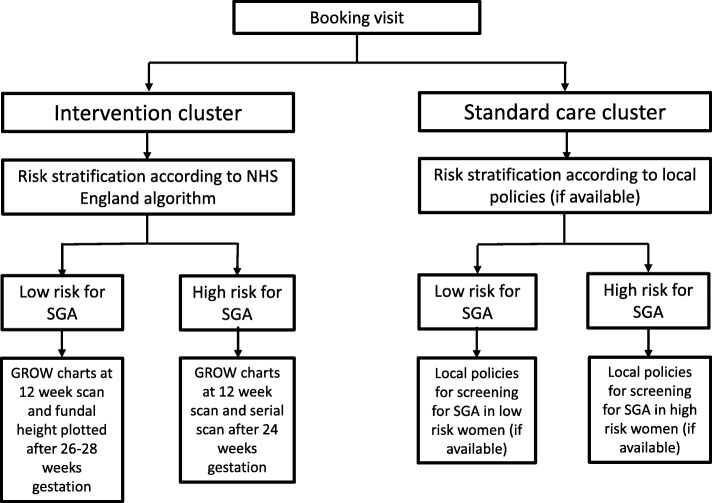
Diagram of individual management within participating clusters

**Fig. 4:**
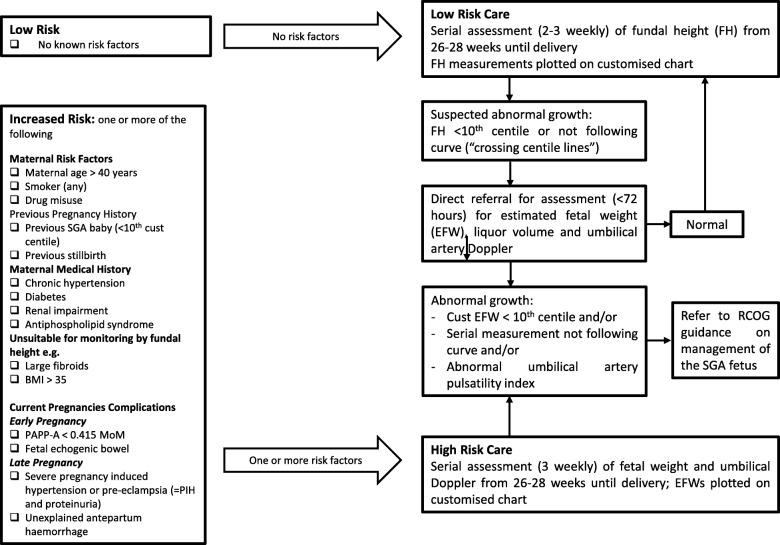
NHS England protocol for screening small-for-gestational-age (SGA) foetuses

The study intervention is implementation of the GAP programme (see Fig. 3) [17]. This includes cascading staff training, adopting or refining evidence-based protocols for SGA detection (Fig. 4), routine monitoring of SGA and detection rates, regular audits of missed cases and ongoing support between the Perinatal Institute and Trusts. Management for women in hospitals randomised to the implementation arm (GAP) is described in Table 1.

**Table 1:** Recommended management of women in clusters randomised to Growth Assessment Protocol (GAP)

Risk assessment for small for gestational age (SGA) (see Fig. 4) and management as per the GAP protocol	
• Women at low risk of SGA will be seen in antenatal clinic, where standardised fundal-height measurements will be performed (2–3 weekly), starting from 26 to 28 weeks	
• For women at high risk of SGA, serial ultrasounds will be recommended, every 3 weeks starting from 26 to 28 completed gestational weeks	
Customised fundal-height and ultrasound charts will be generated in early pregnancy for all women using the gestation-related optimal weight • (GROW) software. Both ultrasound generated estimated fetal weights (EFWs) and fundal heights will be plotted on the customised chart	
• Any deviation from expected progressive growth of fundal height on these charts will raise a recommendation for fetal ultrasound measurement, as will first plots below the 10th centile line	
• Any deviation from expected growth on these charts from ultrasound generated EFW measurements will prompt surveillance according to the Royal College of Obstetricians and Gynaecologists (RCOG) Investigation and Management of the Small-for-Gestational-Age Fetus guidance [25]	

In the standard care arm, women will receive routine care as per their current hospital practice on screening and management of SGA (see Fig. 3). In the absence of GAP, primary screening for anomalies of fetal growth in the UK commonly includes fundal-height measurements that are either plotted onto (non-customised) antenatal growth charts or approximated to the gestational period. Approximation to the gestational period refers to McDonald’s rule, the number of centimetres is expected to approximately equal the gestational age in weeks (± 2–3 cm). The population chart in clinical use in the standard care arm was not pre-specified.

The equation used to calculate the estimated fetal weight based on ultrasound biometry was not pre-specified in the study protocol for either arm of the trial, but Hadlock is the most commonly used in the UK.

*Training and accreditation –* Training in GAP will be provided by the Perinatal Institute, the only provider of GAP and its training, to nominated trainers within each cluster. These trainers will then be expected to disseminate this training to > 75% of multidisciplinary staff engaged in antenatal care within their unit. E-learning and assessment packages, as well as competency certificates, are available to reinforce this. Each cluster will have the uptake monitored via online training logs.

*Audit –* Routine quarterly reporting of SGA rates at birth and its antenatal detection is considered an essential component of the GAP programme. Together with the audit of missed cases of SGA, these feedback mechanisms identify local issues and provide insight on improving performance.

*Support and communication –* As a routine requirement for GAP implementation, Trusts are asked to nominate link persons from each specialty (midwifery, sonography and an obstetric/fetal medicine lead) who will provide local leadership and liaise with the GAP team at the Perinatal Institute.

### Recruitment and allocation

We have already recruited 13 clusters to the trial. The first eight clusters committing to participate were divided into two strata of four clusters each according to their size (number of deliveries during the year 2013–2014). Two further strata (of three and then two) clusters were subsequently defined for clusters that agreed to participate in the study at later dates. For the stratum of size 3 it was randomly determined that two clusters would be allocated to intervention and one to control rather than vice versa, and in all other strata allocation was equally to intervention and control, so that, in total, seven clusters have been allocated to intervention and six to the control. Within-strata allocation to intervention and control was by random permutation using Stata v14 (StataCorp LP, College Station, TX, USA). Due to the nature of the intervention, concealment is not possible.

### Retention and compliance

Regular meetings of GAP leads have been arranged during implementation and were led by the Perinatal Institute to support the implementation of GAP. These meetings support implementation and provide a forum for Trusts to share experiences and solutions. Research meetings with site leads are being held throughout the study period to ensure continued engagement with the research project for provision of data for trial purposes. Regular email correspondence and newsletters are distributed to all cluster sites.

Pre-specified requirements for cluster compliance with GAP comprise: (1) identification of a local multidisciplinary GAP team; (2) a pre-specified proportion of staff who have completed training and (3) confirmation that local guidelines and audit are in line with GAP recommendations. A minimum of 75% staff should receive face-to-face training and be e-learning compliant. Both the Perinatal Institute and the trial team will support non-compliant clusters to discuss strategies to improve compliance with GAP. If these clusters remain non-compliant by the time of data collection, despite attempts to comply, they will still be analysed as randomised to GAP in the intention-to-treat analyses, but the lack of compliance will be acknowledged in other analyses (see the ‘Analysis methods’ section).

Retention and compliance in units randomised to standard care is maintained through regular contact via site visits, newsletter and review of changes in clinical guidelines which may have an impact on the detection of SGA infants (e.g. implementation of the NHS England Saving Babies’ Lives Care Bundle [5]).

### Outcomes

**Fig. 5:**
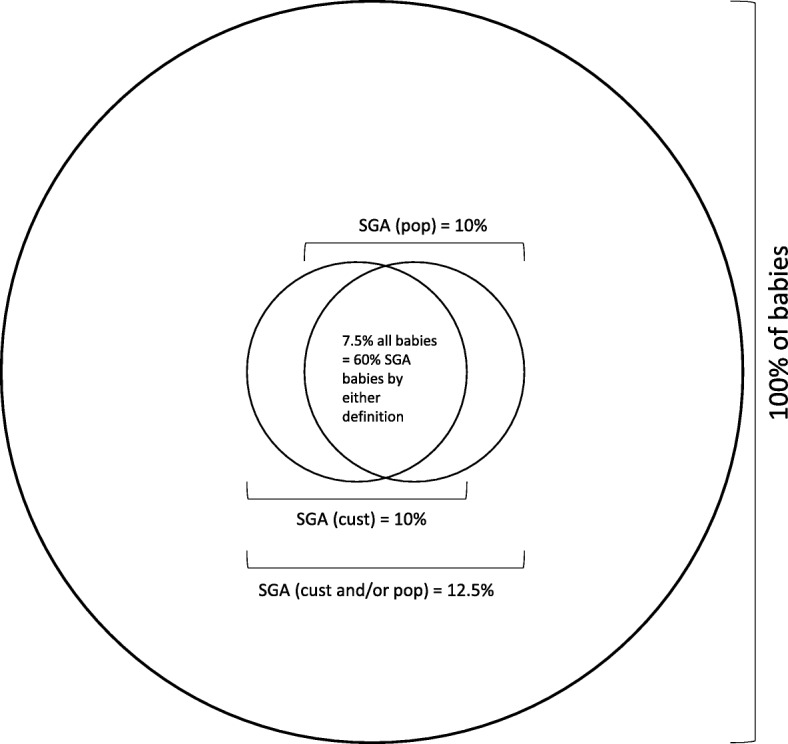
Proportion of small-for-gestational-age (SGA) babies defined by population references, customised standards or both

The primary outcome of this study is antenatal ultrasound detection of SGA (after 24 completed weeks of gestation) in infants who are also SGA at birth. In this trial, we are focussed on the antenatal detection of those infants who weigh less than the 10th centile for gestational age on both population and customised growth charts at birth (Fig. 5). This group of infants will be the denominator (SGA at birth) for the estimation of the detection rate and will be determined using birthweight charts (GROW customised chart and UK90 population chart) [29, 30]. Amongst these infants, the numerator (antenatal ultrasound detection of SGA) will be defined as ultrasound-derived estimated fetal weight < 10th centile by customised charts in the GAP implementation arm and by population charts in the standard care arm [29–31].

The key secondary outcomes are classified according to: clinical outcomes, health-economic outcomes and outcomes derived from process evaluation of the programme implementation; see Table 2 for details. These outcomes will be compared between the two arms of the trial during a pre-specified period of minimum 4 months which will be referred to as the ‘trial outcome period’.

**Table 2:** Key secondary outcomes

Clinical outcomes			Health-economic outcomes	Process evaluation of implementation
Antenatal assessments	Neonatal outcomes	Maternal outcomes		
Rate of antenatal ultrasound detection of SGA at birth by customised standards and by population references. Antenatal clinical detection^a^ of SGA^b^. Analysis of GAP diagnostic test performance (specificity, sensitivity, negative predictive value, positive predictive value)^b^ Ultrasound assessment of SGA using a different threshold, e.g. 5th centile^b^. Growth trajectories (fetal biometry and EFW) and Doppler parameters in the detection of SGA^b^. Comparison of GROW ultrasound charts against standard population charts on classification of fetal growth (small for gestational age, appropriate for gestational age, large for gestational age)^b^	Basic parameters: Gestational age at birth Birthweight Head circumference	Antenatal: Length of stay in hospital	Number of ultrasound scans after 24 weeks Antenatal clinic/antenatal day unit activity Rates of induction of labour Rates of caesarean sections Length of maternal and neonatal stay Admissions and average length of stay in NICU/SCBU	Proportion of staff trained, staff assessed and women assessed with GAP programme Adherence to SGA risk stratification and management protocols Adherence to missed-case analysis Evaluation of acceptability and feasibility of intervention to staff and women, contextual barriers and facilitators, practice in control sites Organisational impact and unintended consequences
	Condition at birth: 5-min Apgar score < 7 Arterial cord pH < 7.1 Any respiratory support given at delivery	Intrapartum: Induction of labour Mode of delivery (including caesarean section rates) Postpartum haemorrhage Rates of 3rd or 4th degree perineal tear		
	Neonatal admissions: Length of stay at each neonatal level of care			
	Neonatal morbidity: Major neonatal morbidity (any of neonatal brain injury, receipt of supplemental oxygen at 28 days of age, Bell stage 2+ necrotising enterocolitis, culture-positive sepsis, retinopathy requiring ophthalmic intervention) Minor neonatal morbidity (any of: hypothermia, hypoglycaemia, nasogastric tube feeding)	Postnatal: Length of stay in hospital Breastfeeding at discharge		
	Perinatal loss: Antepartum or intrapartum stillbirth Neonatal death (early or late) Death before neonatal discharge (after 28 days of birth) Cause of death			

*Abbreviations*: *EFW* estimated fetal weight, *GAP* Growth Assessment Protocol, *GROW* gestation-related optimal weight, *NICU* neonatal intensive care unit, *SCBU* special care baby unit, *SGA* small for gestational age

^a^Clinical detection of SGA is defined as ‘antenatal acknowledgement that the foetus is expected to weigh below the 10th centile at birth, by charts appropriate to the study arm’

^b^These secondary outcomes will not be reported in the first clinical paper; please see the section on ‘Presentation and publication strategy’

### Sample size – power calculation

A minimum target sample size of 12 clusters (six per arm) was set based on information collected during protocol development. First, we assessed that the mean births per cluster per year in a sample of London maternity Trusts likely to participate in the trial was 5053. We assumed a 10% rate of SGA by a single definition. Pooled estimates from previous studies suggest that 75% of SGA infants defined by customised standards are also SGA by population references and vice versa [19, 22, 32, 33]. Therefore, we estimate a SGA rate by either definition (customised or population) of 12.5% and 60% of these babies (7.5% of total sample) will be SGA by both definitions (Fig. 5).

During the trial outcome period (minimum of 4 months) we anticipate a mean of 42 babies to be SGA by customised standards only; 42 SGA by population references only; and 126 SGA by both definitions per cluster based on the assumptions above (see Distribution – Table 3). Further details of this assumptions are provided in Additional file 1: Table S1-S2. However, we have explored the impact on power of fewer babies meeting both SGA definitions, defined as a mean of only 84 SGA babies per cluster in the unlikely extreme scenario where the number of infants in the overlap is reduced by a third. Published reporting using detection during standard antenatal care suggest that, regardless of whether population or customised standards are used to define SGA at birth, around 20% of SGA births are detected antenatally [11, 12, 17]. For the intervention arm (GAP programme), we anticipate an improvement in our primary outcome from 20 to 33%.

**Table 3:** Distribution of small for gestational age (SGA) and expected detection rates

		Number of small-for-gestational-age (SGA) neonates/10,000 births according to pooled estimates of previous studies [19, 22, 32, 33]		
		By population reference only	By both population reference and customised standards (*primary outcome*)	By customised standards only
Total observations with SGA infants		250	750	250
Detection – standard care arm	%	20%	20%	16%
	*N*	50	150	40
Detection – implementation arm (Growth Assessment Protocol; GAP)	%	12%	33%	33%
	*N*	30	250	83

We were unable to identify reports of an intra-cluster correlation coefficient for detection of SGA; therefore, a coefficient of the most approximate outcome (fetal growth restriction) was used (0.019) [34]. A cluster size of 126 SGA infants (by customised standards and population references) and six clusters in each arm provides 84% power to demonstrate superiority of GAP at the 5% significance level (two-sided test) for our primary outcome. In the alternative unlikely scenario of only 84 SGA infants by both definitions per cluster the design provides 79% power. Power calculations were performed using the user-written programme *clustersampsi* for Stata [35].

We also performed power calculations for two secondary outcomes. This sample size will also provide 91% power to demonstrate a superiority of GAP in detecting SGA defined by customised standards (increase in detection from 19 to 33%) and results in over 90% power to demonstrate non-inferiority of the intervention for the ultrasound detection of SGA by population references (increase from 20 to 28%, considering an example non-inferiority margin of 5%).

### Data collection and management

Most data will be acquired from routine hospital systems. Data obtained either manually from clinical notes, or electronically from hospital maternity and neonatal databases will be entered/uploaded into the study database. Data will be pseudonymised and linked to a study identification number at each site before the pseudonymised data is sent electronically to the trial team. The key connecting participant details to study identification number will be password-protected and kept locally at study sites on NHS networks.

Data collected during the study includes clinical data from the period prior to implementation (baseline data) and during the trial, together with rates of training compliance. We will use a calculator provided by the Perinatal Institute (GROW calculator) [30], which determines customised birthweight standards. A review of the fidelity of GAP use will inform data monitoring and the implementation evaluation, and clinical and service use data will be collected for a 4–6-month period after full GAP implementation, to assess primary and secondary outcomes of this study.

For the process evaluation of implementation, quantitative data (including proportion of staff trained/assessed, women managed with the GAP programme, missed-case audit) will be collected. Research staff will also perform an audit of selected patient notes (women in whom SGA was missed) to check for adherence to risk stratification and management protocols.

Qualitative, semi-structured interviews with purposively sampled key staff/stakeholders will be performed to assess barriers and facilitators of implementing this complex intervention. In addition, local policies or guidance for antenatal management of associated conditions, or interventions, will be collated centrally (e.g. guidance on reduced fetal movements, policy for routine third-trimester ultrasound or diabetes in pregnancy).

### Analysis methods

#### Statistical analysis

The analysis will acknowledge the clustering of individual participants by centre. Due to the modest number of clusters the analysis will be performed using a cluster-summary statistic approach, i.e. calculating the proportion with the primary outcome for each cluster and comparing these values between intervention and standard care arms and presenting 95% confidence intervals for the average difference. Adjustment will be made for key characteristics of the individual participants, e.g. age, parity, ethnicity to account for imbalance between arms that might occur from the randomisation of only a modest number of clusters. An adjusted cluster summary value of the primary outcome will be generated for each cluster for both the trial period and the baseline (i.e. pre-trial) period. Then, an analysis of covariance (ANCOVA) analysis will be conducted to compare intervention and control clusters accounting for differences at baseline. The primary analysis will be on a modified intention-to-treat basis, in which any Trusts in the intervention arm that did not contact the GAP provider to initiate training and implement the intervention in the study period due to changes in local strategy are excluded, since such changes are not considered informative concerning how GAP would have performed in the Trust. In addition, we will conduct an intention-to-treat analysis including all Trusts as randomised. This sensitivity analysis will not be influenced by potential differences between a Trust that is randomised to the GAP arm but does not register to use it, compared to those that do use it, which could confound the effect estimate. A further secondary analysis will be conducted under a per-protocol approach restricting analysis of the intervention arm to clusters that complied with GAP (as defined in the ‘Retention and compliance’ section).

Subgroup analyses will be performed to assess the intervention effect amongst women who have had full exposure to GAP (charts generated prior to 24 weeks’ gestation) and those women who have only had late exposure (e.g. those women who transfer care to the hospital after 24 weeks’ gestation). A detailed analysis plan will be prepared before the analysis begins.

#### Health economic evaluation

A cost-effectiveness analysis will be carried out on the primary clinical outcome (detection of SGA). This will evaluate probabilistically whether the intervention is unequivocally cost-effective compared to standard practice (e.g. less costly and more effective), less cost-effective (e.g. more costly and less effective) or whether GAP achieves improved rates of SGA detection at greater cost compared to standard practice.

The economic evaluation will draw on patient-level clinical resource use (e.g. use of ultrasound) with data extracted from the hospital records combined with data on the primary clinical outcome to evaluate the cost-effectiveness of GAP. National and locally applicable unit costs will be used to cost resource-use identified for each patient, and subsequently to evaluate the difference between the pregnancy and neonatal care costs, between the intervention and control sites. We will also evaluate the impact of costs relating to additional activities required for successful implementation of the GAP protocol on conclusions regarding cost-effectiveness, including the cost of training clinical teams and on-going costs associated with monitoring adherence to the GAP protocol.

The economic evaluation will be carried out from the NHS hospital (NHS Trust) perspective and will, therefore, exclude measurement of wider resource use linked to activities that are not undertaken by Trust maternity or neonatal care teams, e.g. use of community-based primary care services. Resource use and cost impacts will only be included in the evaluation up to the point of postnatal inpatient discharge.

#### Analysis of implementation

The process evaluation aims to understand how the GAP intervention works in practice, by examining implementation, mechanisms of impact, and contextual factors. Implementation of the intervention will be evaluated via a mixed-methods approach drawing on the UK Medical Research Council framework for trials of complex interventions [36, 37]. Following Steckler and Linnan’s approach [38], the key dimensions of implementation to be analysed include those detailed in Table 4.

**Table 4:** Dimensions of implementation for analysis [38]

Dimension	Description
Implementation process	The structures, resources and mechanisms through which delivery is achieved
Fidelity	The consistency of what is implemented with the planned intervention
Adaptations	Alterations made to an intervention to achieve better contextual fit
Dose	How much intervention is delivered
Reach	The extent to which a target audience encounters the intervention
Mechanisms of impact	The intermediate mechanisms through which intervention activities produce intended (or unintended) effects

We will describe the intervention and the mechanisms through which it is expected to produce change in a specific context using the Template for Intervention Description and Replication (TIDieR) guidance [39] and produce a logic model which informs data items for the evaluation of implementation.

For process evaluation of implementation, descriptive quantitative information on fidelity, dose and reach will enable us to consider more detailed modelling of variations between participants or sites in terms of factors such as fidelity or reach (e.g. are there ethnic or socioeconomic biases in who is reached?).

For evaluation of acceptability and feasibility of intervention to staff and women, contextual barriers and facilitators and organisational impact, gathered through qualitative data collected at six intervention sites. We will also undertake qualitative interviews at control (delayed implementation) sites, to explore whether measures similar to the GAP intervention have been adopted, perhaps as part of the NHS England ‘Stillbirth Care Bundle’.

### Data monitoring

#### Data Monitoring Committee

A joint Trial Steering and Data Monitoring Committee (TSC/DMC) has been formed. It will meet halfway through the trial to review the progress of the trial, any adverse events and the proposed analysis plan. At the end of the trial, it will meet to review the proposed trial outputs. There is no planned interim analysis of the trial outcomes, and outcome data will only be analysed at the end of the trial.

#### Monitoring for potential harms

The non-medicinal intervention being tested in this trial is not expected to have side effects and is already widely adopted across the UK. Nevertheless, we shall monitor for harms due to the intervention, or its implementation within the trial, as for the sites concerned this is a new method of patient care. The intervention comprises measurement of fundal height and prompt referral for ultrasound where needed. Side effects of fundal-height measurement may consist of maternal discomfort due to the semi-recumbent position required and discomfort due to increased skin sensitivity. Ultrasound is a mechanical wave and can theoretically increase the temperature in the studied tissue. The Doppler ultrasound uses higher energy and focusses on a smaller volume of tissue, resulting in greater changes in temperature. In a clinical obstetric scenario, however, the increase in temperature is less than 1 °C, which is not considered clinically significant. The World Health Organisation performed a systematic review of 61 publications on the subject and reported no association with adverse maternal, fetal and neonatal outcomes [40]. Both components of the intervention are used in different levels in routine care; therefore, any of the above cannot be strictly assigned to the intervention.

Adverse events may occur and may be attributed to incorrect use of the GAP programme. As an example, mistakes in the manual plotting of fundal height on any chart could lead to missed referrals and ultimately the potential for a serious adverse event (SAE). The audit of missed SGA cases and the review of all stillbirth cases will be performed locally in each cluster as recommended by GAP. Any event associated with misuse of GAP will be reported as an adverse event in the trial. Any serious adverse events recognised by the sites because of the intervention (which result in death, life-threatening conditions or prolonged hospitalisation) must be reported to the sponsor within one working day of the investigator becoming aware of the event. Both the site clinical team and the sponsor should investigate the SAE for causality. The trial team will also record instances of concerns raised by either staff or participants at intervention sites relating to the intervention or how it is implemented.

### Presentation and publication strategy

The primary and selected secondary maternal and neonatal outcomes will be published together in the first paper (see Table 2). Further papers will be submitted for publication for the detailed health-economic analysis, implementation evaluation, analysis of alternative methods of measuring detection of growth restriction and additional subgroup analyses.

## Discussion

This study will provide the first evidence from a RCT into the effect of implementing the GAP programme, compared to standard care, on the identification of SGA measured by ultrasound scan. The study is powered for detection of SGA babies, and not for stillbirth rates. Although a study to show differences in stillbirth is ultimately desirable, it is unfortunately not feasible to power such a trial [41] due to the infrequency of this event and hence very large sample size required. It is widely recognised that antenatal detection of SGA is associated with reduction in stillbirth rates [10].

The primary outcome for this trial has been chosen following consensus agreement from the co-investigators and independent reviewers. It is recognised that there is an overlap between SGA defined by customised and population standards. The group of infants meeting criteria for both definitions are at the highest risk of morbidity/mortality [19]. Detection of these babies is crucial. Focussing on this overlapping group addresses the issue that GAP was not developed to detect SGA by population standards, and vice versa.

Customised standards have been criticised because some factors (namely maternal weight and ethnicity) might contribute to differences in birth weights due to pathological, rather than physiological, mechanisms [32, 42, 43]. The Perinatal Institute recommends the adjustment for maternal weight based on their report that, in women with a Body Mass Index (BMI) within the normal range (20–25 kg/m^2^), variation in maternal weight was not associated with increased risk of perinatal mortality. In women with BMI > 25 kg/m^2^, the rate of perinatal mortality was correlated with SGA using customised standards but not population references [44]. However, cause-specific perinatal mortality may differ by maternal BMI. The influence of ethnicity is more complex due to the known association between ethnicity and socioeconomic deprivation [45–47], which is associated with antepartum stillbirth risk [48, 49]. The INTERGROWTH-21st study recruited low-risk, well-nourished women of optimal health, education and socioeconomic status in urban sites with low levels of pollutants [50] and showed similarities in fetal growth (as assessed by head circumference) and newborn length in those pregnancies studied across eight international and multi-ethnic sites. In contrast, work using the Canadian Stillbirth Registry reported that when using population standards, women of Chinese and South Asian ethnicity have higher rates of SGA, when compared to native ethnicities, but lower rates of perinatal mortality. In the same group, the rate of SGA was lower and concordant with the prevalence of perinatal mortality when neonates were assessed using customised, rather than population growth, standards [51]. More recently, the World Health Organisation has published new fetal growth charts that do acknowledge differences in fetal growth between women of different ethnicities, heights, weights, ages and parity [52]. According to this evidence, adjustment for ethnicity may improve the detection of SGA infants at risk of morbidity and mortality, irrespective of any indirect adjustment for the pathological influences such as socioeconomic conditions.

In the UK, there is a high-level ambition to reduce the rate of stillbirths, arising from the RCOG and the Department for Health [4, 6]. Maternity clinical networks and local maternity systems are currently recommending several interventions aimed at reducing rates of stillbirth in the UK [5]. One theme of the national stillbirth recommendations is reduction of SGA-related stillbirth by improved detection of SGA. The GAP programme is the most widely adopted of the available potential solutions, leaving few available sites for recruitment into this trial. We therefore also face the last opportunity to conduct a prospective randomised trial of customised growth charts and the GAP protocol in the UK. Finally, other national initiatives to reduce stillbirth include policies to support smoking cessation, improve intrapartum fetal monitoring and increase reporting of reduced fetal movements [5]. Uptake of smoking cessation support is anticipated to impact upon rates of SGA infants, which could affect analysis of our primary outcome, and all initiatives may impact upon several of the secondary outcomes in this trial. Hospitals recruited to both arms of the trial are under external pressure to adopt some of these policies. We are collecting information on all policies introduced which are independent of the intervention during the trial period to evaluate any potential effect of contamination.

Regarding the health-economic analyses, we have considered the usefulness of reporting the costs from a Trust or health service perspective. The current UK payment system uses tariffs, according to a low-/high-risk stratification of individual patients, rather than crude costs for work done. Whilst this will allow us to calculate the cost of the GAP programme to the health service (by estimating any changes to the likely tariff distribution because of GAP), it does not necessarily translate into financial cost to the hospitals themselves from providing resources to implement GAP, or from the implications of implementing GAP on the patients/resources (by affecting rates of SGA diagnosis). This justifies our approach of reporting the health-economic analysis from a Trust perspective.

Regarding the implementation analysis, we anticipate variation between clusters in GAP implementation, including local adaptations to suit available resources (e.g. number of sonographer lists, and the financial resources required to implement the programme). Such variations will need to be placed in context when assumptions are made about external validity. Nevertheless, the implementation study should produce documented evidence of such differences, and by examining these in relation to the logic model produced for the study, we hope to extend understanding of how variations in implementation might affect the impact of GAP in real-world situations.

Overall, we aim to provide high-quality evidence, through a RCT, of the performance and service provision requirements of the GAP protocol. This may have the potential to inform national policy decisions on methods to reduce the rate of stillbirth.

## Trial status

The first eight cluster sites were randomised to either the intervention or control arm on 3 November 2016. A further three sites were randomised in December 2016 and the final two sites randomised in July 2017. Baseline audit and training procedures were then required prior to the use of the intervention in the active clusters. The first cluster sites began to use the intervention in October 2017 and we anticipate that the intervention will continue to be applied to women attending for maternity care at those sites, with the last women to be included in the data collection likely to have the intervention applied in early July 2018. Data will be collected on all babies being born between 1 June and 31 November 2018.

## Additional file

Additional file 1: **Table S1.** Standard Protocol Items: Recommendations for Interventional Trials (SPIRIT) Checklist (2013): recommended items to address in a clinical trial protocol and related documents. **Table S2.** Distribution of small-for-gestational-age (SGA) and expected detection rates. (DOCX 41 kb)

## Abbreviations

ANCOVA: Analysis of covariance
BMI: Body Mass Index
DESiGN: DEtection of the Small for Gestational age Neonate
DMC: Data Monitoring Committee
EFW: Estimated fetal weight
GAP: Growth Assessment Protocol
GROW: Gestation-related optimal weight
ISRCTN: International Standard Registered Clinical/soCial sTudy Number
KCL: King’s College London
MBRRACE UK: Mothers and Babies: Reducing Risk through Audits and Confidential Enquiries across the UK
NHS: National Health Service
NICU: Neonatal intensive care unit
RCOG: Royal College of Obstetricians and Gynaecologists
RCT: Randomised controlled trial
SAE: Serious adverse event
SCBU: Special care baby unit
SGA: Small for gestational age
TSC: Trial Steering Committee
UK: United Kingdom
